# Development and evaluation of a new multidimensional oral health indicator

**DOI:** 10.3389/froh.2025.1634245

**Published:** 2025-09-30

**Authors:** José João Mendes, Rui Santos, Patrícia Lyra, Ana Sintra Delgado, Vanessa Machado, João Botelho, Luís Proença

**Affiliations:** Egas Moniz Center for Interdisciplinary Research, Egas Moniz School of Health and Science, Almada, Portugal

**Keywords:** oral health related quality of life (OHRQOL), dental caries, periodontitis, oral health value, patient reported clinical outcomes

## Abstract

**Aim:**

To develop a new multidimensional oral health indicator (MOHi) to ascertain and characterize overall oral health status.

**Materials and Methods:**

MOHi was developed using data from first-time patients (*N* = 1,034) attending a university dental clinic over an 18-month period. Participants completed the Oral Health Value Scale (OHVS), the Oral Health Impact Profile-14 (OHIP-14) and self-reported periodontal status questionnaires. Caries experience through Decayed, Missing and Filled Teeth (DMFT) index, was combined with radiographs. MOHi was built as a linear combination of the normalized OHVS, OHIP-14 and DMFT scores, in a continuous scale from 0 to 3, with higher scores representing a more degraded oral health condition. MOHi was evaluated according to sociodemographic, behavioral characteristics, and periodontal self-reported status. We compared MOHi mean values with t- Student's test and ANOVA one-way. Stepwise multivariate logistic regression methodologies modelled MOHi and identified significant predictors. Model performance was evaluated by ROC/AUC analysis.

**Results:**

MOHi had a normal distribution adequacy (ranging 0.29–2.47), with an average of 1.22 (±0.41). Significantly higher MOHi scores were found in patients with self-reported periodontitis (*p* < 0.001), former/active smokers (*p* < 0.001), elementary/middle education level (*p* < 0.001), employed/retired (*p* < 0.001), age >= 45 years (*p* < 0.001) and married/divorced/widowed (*p* < 0.001). The final reduced logistic regression model identified age (OR = 1.05), self-reported periodontitis (OR = 1.94), sex—female (OR = 1.80), smoking status active/former (OR = 3.12/OR = 1.62), education level—elementary/middle (OR = 2.94/OR = 2.27) as predictor conditions towards a more degraded oral health condition (AUC = 0.81).

**Conclusion:**

MOHi has shown to be a promising tool to comprehensively characterize the overall oral health status.

## 1 Introduction

Health behaviors are influenced by factors, including psychological aspects, socio-economic status, emotional state, attitudes, beliefs, education, social context, health policy, and access to oral health care (1). In oral health, cultural and care quality available in a community shape views on oral health and use of oral health services (2). At the core are oral health values (OHV), reflecting how individuals prioritize and allocate resources to oral health care. These values show considerable variability, influencing the prioritization process on health behaviors (3).

Oral health–related quality of life (OHRQoL) captures the extent to which oral conditions affect daily functioning, social interactions, and well-being (4). Poor oral health deteriorates oral health-related quality of life (OHRQoL) (3), a self-reported measure of how patient's quality of life and life satisfaction are adversely affected (5, 6). Defining OHRQoL is challenging (6), and may primarily reflect the views of clinicians and not adequately consider patients’ OHV (7). This highlights the need for integrating OHV that considers each patient's unique perspective on oral health and how it truly affects them. Emerging evidence suggests that OHV may be key determinants of treatment-seeking behaviors, alongside other health-related scores (8). Oral health problems disproportionately affect low-income groups, minorities, individuals with disabilities, women (despite their higher frequency of dental visits compared to men), edentulous individuals, and older adults (8, 9). These disparities suggest lower OHV correlate with increased risks of deteriorating oral health outcomes.

Integrating patient reported outcome measures (PROMs) (OHRQoL and OHV) with clinical data into a comprehensive assessment framework could capture subjective perceptions and objective measures, providing a nuanced evaluation of oral health. In addition, the accessibility across clinical settings could be a valuable public health and epidemiology instrument, aiding in identifying at-risk populations and informing targeted interventions. This multidimensional perspective ensures oral health assessments extend beyond clinical findings, incorporating individual experiences and values to support more equitable and patient-centered care strategies.

Thus, this study aimed to develop a new multidimensional oral health indicator (MOHi) to ascertain and characterize the overall oral health status of an individual, by combining OHRQoL, OHV and clinical information.

## 2 Methods

### 2.1 Study design, setting and participants

For the present study, data from a cohort of patients followed at a Portuguese university dental clinic (Egas Moniz Dental Clinic, Almada, Portugal) was used. Data collection followed a consecutive sampling of incoming participants, from January 2022 until June 2024, that seek a first appointment. The study was approved by the Egas Moniz Ethics Committee (1050/2022) and all participants gave their informed consent. The study is reported according to the Transparent reporting of a multivariable prediction model for individual prognosis or diagnosis (TRIPOD) for model development (10).

### 2.2 Eligibility criteria and sampling

To be included in this study, participants had to: have 18 years of age or older; being able to read, understand, and sign the informed consent form; and were seeking initial triage at the university dental clinic. Participants were invited to participate voluntarily and anonymously. Given the lack of studies integrating clinical oral conditions with patient reported outcomes, we did not estimate a minimum sample size and opted for study design based on a consecutive random sample obtained over a pre-defined timescale (18-month).

### 2.3 Variables

#### 2.3.1 Outcome variables

Caries experience was measured using the Decayed, Missing, and Filled Teeth (DMFT) index. To calculate the DMFT score, a clinical examination of the teeth was conducted and registered on a computerized chart. The final score is often used in research studies to compare the dental health of different populations and evaluate the effectiveness of dental health programs (11).

The screening of periodontal status was carried out using a self-reporting approach previously validated using thirteen questions, of which two have an Area Under the Curve (AUC) of 0.8 of predictive ability (12) the number of tooth lost observed clinically. The remaining questions included information regarding gum and teeth health, loose teeth, bone loss, tooth appearance, and the use of dental floss and mouthwash (12, 13).

#### 2.3.2 Exposure variables

Data were collected through a self-reported questionnaire on sociodemographic characteristics and behavioral aspects. This questionnaire was administered prior to taking panoramic radiographic and clinical oral observation. Overall, the information collected included sex, age, marital status (categorized as single, married/cohabiting, divorced or widowed), level of education [categorized as “elementary” [9 years schooling], “middle” [secondary school or vocational training, started/completed] and “higher” [college/university level]], occupation (categorized as student, employed, unemployed or retired), medical conditions. Smoking habits was categorized according to the NHANES methodology (14) as non-smoker (never smoked or smoked less than 100 cigarettes in life), ex-smoker (smoked less at least 100 cigarettes in life and currently does not smoke) and active smoker (smoked at least 100 cigarettes in life and currently smokes). We also questioned how many cigarettes per day and for how long.

To measure the priority and relevance of oral healthcare for each participant, the Portuguese-validated version of the OHVS was used (3, 15). The Oral Health Value Scale (OHVS) is a comprehensive assessment tool that evaluates multiple domains of oral health to comprehensively understand a patient's oral health status (3), recently validated to Portuguese (15). This instrument consists of 12 items that cover professional dental care (items 4, 8 and 11), appearance and health (items 3, 7 and 12), flossing (items 2, 5 and 10), and retaining natural teeth (items 1, 6 and 9). Each question is rated on a 5-point Likert scale, with responses ranging from “strongly disagree” to “strongly agree”, allowing for a nuanced understanding of the patient's responses and enabling the dental professional to identify and address any potential issues in a timely manner.

The Portuguese-validated version of the Oral Health Impact Profile (OHIP-14) (16) was used to evaluate the impact of oral health on the quality of life of each study participant. The OHIP-14 consists of 14 questions that are used to appraise to which extent oral health problems interfere with various aspects of a patient's life, including physical pain, physical disability, psychological discomfort, social discomfort and others (5). The mentioned questionnaire discloses how often the individual experienced such a situation is rated on a 5-point scale for each question (never, hardly ever, occasionally, often, very often) (5, 16).

### 2.4 Data analysis and statistical methodologies

The proposed index (MOHi) was developed under the guidelines of the “Handbook on Constructing Composite Indicators: Methodology and User Guide” (17). MOHi was developed as a composite multi-dimension instrument, being built as an equally weighted linear combination of the normalized OHVS, OHIP-14 and DMFT scores. It is expressed in a continuous scale from 0 to 3, with increasing values progressively representing a more degraded oral health condition. After validation and optimization, the MOHi distribution, dynamics and sensitivity was evaluated, not only as a function of patient sociodemographic and behavioral characteristics, but also when considering the individual periodontal (self-reported) status.

Data analyses included descriptive, inferential and modelling procedures, which were performed using IBM SPSS Statistics v.30 software (Armonk, NY, USA). Continuous data were expressed as mean and standard deviation (SD), while categorical variables were expressed as frequency and percentages (%). For continuous variables, the adequacy to normal distribution was evaluated prior to inferential inter-group comparisons (t-Student's test and ANOVA one-way were applied). Stepwise multivariate logistic regression methodologies were used to model MOHi data and identify significant predictors towards a degraded oral health condition (MOHi score >= 1.5). An alternative model was performed with DMFT as a single item, with a cutoff of 14 or higher (18). These two reduced models were compared to verify the comprehensiveness of predictors and performance. Performance was evaluated by Receiver Operator Curve (ROC)/Area under the Curve (AUC) analysis. A significance level of 5% (*p* < 0.05) was used for all statistical inferential analyses.

## 3 Results

### 3.1 Study sample and characteristics

**Figure 1:**
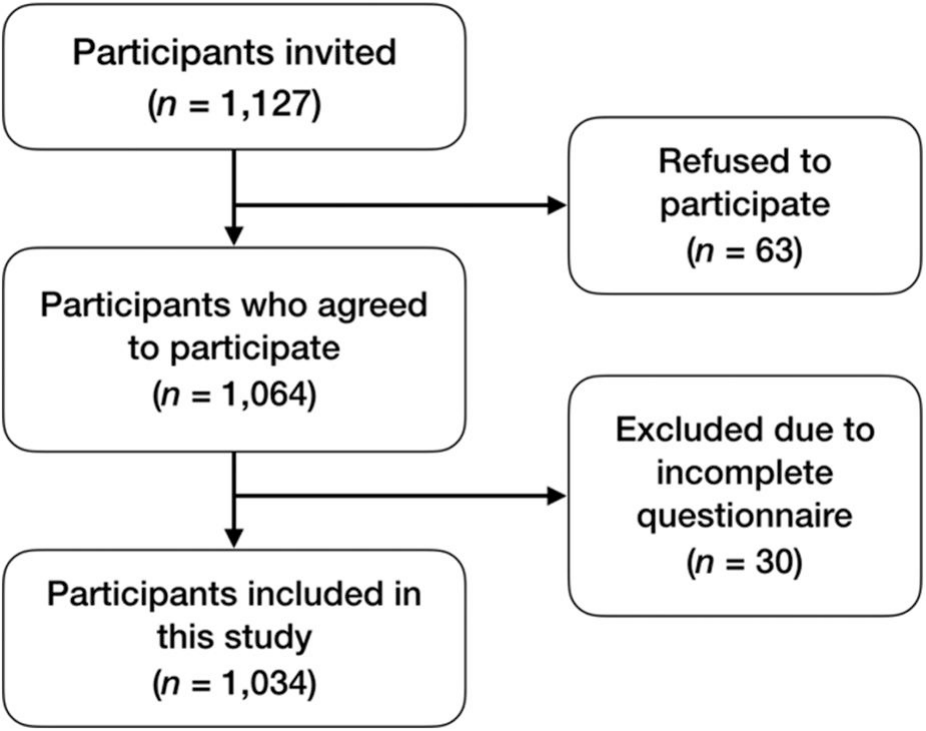
Flowchart of patient inclusion. Data describes the steps involved in the inclusion of the final sample with the number of patients and respective reasons.

For this study, 1,127 participants were invited to participate. A total of sixty-three refused to participate and undergo clinical observation, thirty had incomplete questionnaire responses, resulting in a final sample of 1,034 participants (Figure 1). The characteristics of this population are detailed in Table 1). This sample had a majority of female participants (58.1%), with a mean age of 46.8 (±18.6) years. Most participants were employed (67.9%), with either middle (33.5%) or higher education (33.8%), and 24.7% of active smokers. The average caries experience was 12.8 (±8.4) teeth, with an average of 6.3 (±7.5) missing teeth. About 9.0% of participants had severe tooth loss. Overall, 42.0% of participants had periodontitis according to their self-report. When estimating the oral health value, participants had a mean score of 31.1 (±6.3), and whose percentage scores 64.8% (±13.2). The average OHRQoL was 12.1 (±12.0) and most participants reported a frequently affected quality of life (57.8%).

**Table 1:** Participants sociodemographic and behavioural status (*N* = 1,034).

	Overall (*N* = 1,034)
Age (years), mean (SD)	46.8 (18.6)
Sex, % (*n*)	
Females	58.1 (601)
Males	41.9 (433)
Employment status, % (*n*)	
Student	11.2 (116)
Employed	67.9 (702)
Unemployed	4.0 (41)
Retired	16.9 (175)
Marital status, % (*n*)	
Single	38.8 (401)
Married	45.1 (466)
Divorced	11.6 (120)
Widowed	4.5 (47)
Education, % (*n*)	
Elementary	32.7 (338)
Middle	33.5 (346)
Higher	33.8 (350)
Smoking habits, % (*n*)	
Never	55.3 (210)
Former smoker	20.0 (76)
Active smoker	24.7 (94)
DMFT Index, mean (SD)	
Total	12.8 (8.4)
D	3.2 (3.6)
M	6.3 (7.5)
F	3.4 (3.9)
Severe tooth loss (<10 remaining teeth), % (n)	9.0 (93)
Self-reported periodontitis, % (*n*)	42.0 (434)
OHVS, mean (SD)	31.1 (6.3)
OHVS (%), mean (SD)	64.8 (13.2)
OHIP-14, mean (SD)	12.1 (12.0)

### 3.2 Patient reported outcomes (PROs) and caries experience

The distribution characteristics indicate OHVS and DMFT scores were relatively symmetrically distributed with slight platy kurtosis (Table 2), and OHIP-14 scores were more positively skewed, highlighting a tendency for a larger number of participants to report lower impact levels on their oral health-related quality of life.

**Table 2:** Distribution characteristics of PROs (OHVS and OHIP-14) total scores and caries experience (DMFT) for the included participants (*N* = 1,034).

	Mean (SD)	Median	Min.—Max.	Skewness	Kurtosis
OHVS	31.1 (6.3)	30.0	15–48	0.361	−0.497
OHIP-14	12.1 (11.9)	9.0	0–54	1.026	0.242
DMFT	12.8 (8.4)	12.0	0–32	0.292	−0.763

### 3.3 Development and optimisation of the MOHi

**Figure 2:**
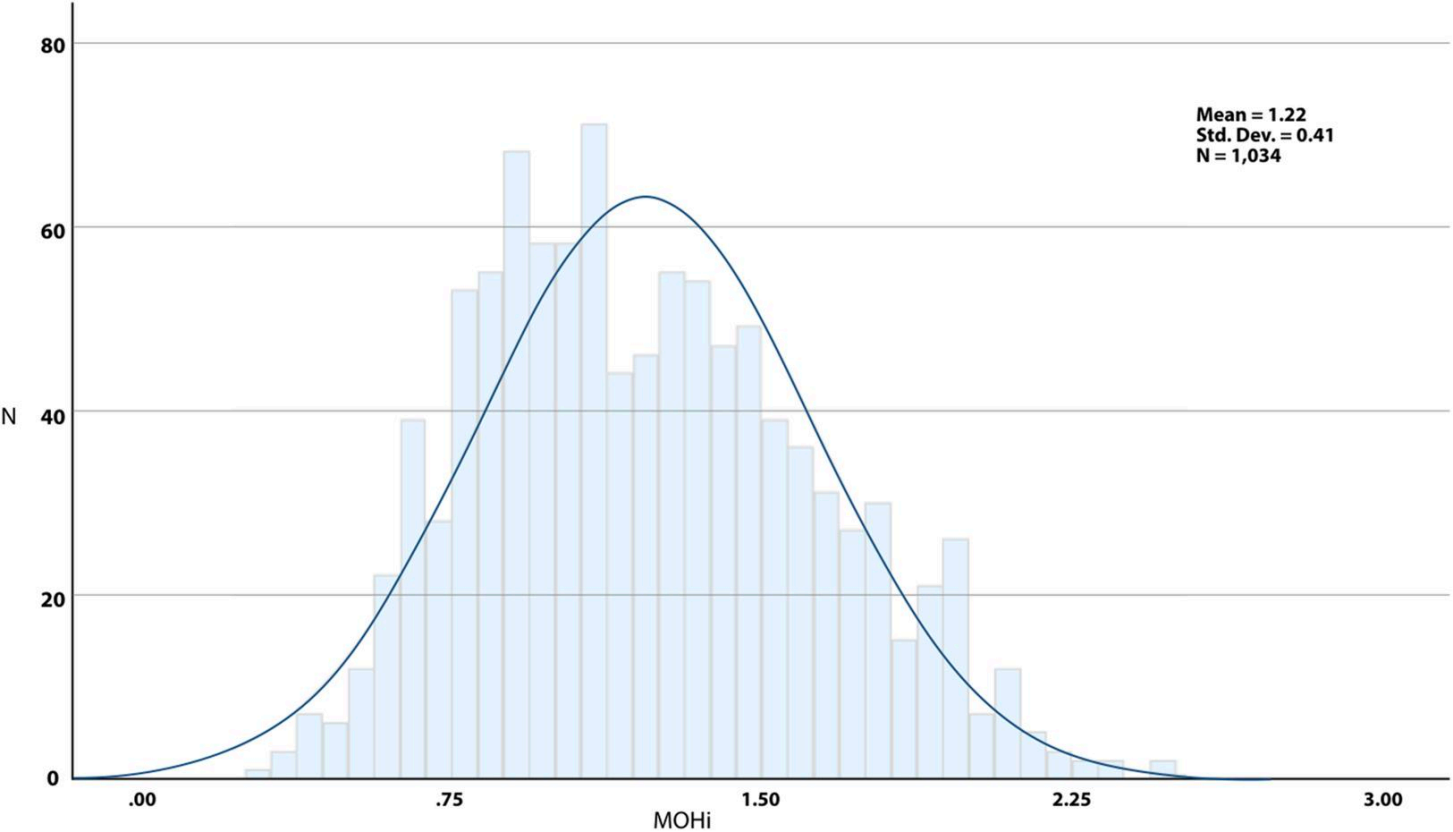
Overall distribution of MOHi values.

The new score composite regarded three normalized algebraic components (Table 3). The MOHi score distribution is fairly symmetrical with a slight right skew (Figure 2 and Table 4), suggesting that most participants score around the mean, but some report higher values.

**Table 3:** Composition of the MOHi score (^a^), in respect to its three normalized algebraic components.

	(A)	(B)	(C)
MOHi = A + B + C	1-[(OHVS score-12)/48]	(OHIP-14 score)/56	(DMFT)/32

a MOHi range: 0–3; MOHi minimum: 0, (for OHVS score=60; OHIP-14 score=0; DMFT=0); MOHi maximum: 3, (for OHVS score=12; OHIP-14 score=56; DMFT=32).

**Table 4:** Characteristics of the MOHi score distribution (*N* = 1,034).

	Mean (SD)	Median	Min.—Max.	Skewness	Kurtosis
MOHi score	1.22 (0.41)	1.18	0.29–2.47	0.351	−0.482

### 3.4 MOHi vs. sociodemographic/behavioural characteristics and self-reported periodontal status

The MOHi scores varied significantly across self-reported periodontal status and sociodemographic and behavioral factors (Table 5). Participants with periodontitis, older adults, individuals with lower education levels, smokers, retirees, and those who were divorced or widowed exhibited higher MOHi scores, indicating poorer oral health outcomes (*p* < 0.001 for all). A distinct age-related trend was observed, with MOHi scores rising from 1.02 in individuals aged 18–44 to 1.47 in those 65 and older. Additionally, lower educational attainment and current smoking habits were linked to poorer outcomes. Although women exhibited slightly higher MOHi scores compared to men, this variance did not reach statistical significance (*p* = 0.066). These results highlight the substantial impact of factors such as age, education level, tobacco use, and socioeconomic conditions on individuals’ perceptions and outcomes related to oral health.

**Table 5:** MOHi mean values comparison as a function of self-reported periodontal status and sociodemographic/behavioural characteristics (*N* = 1,034).

Variable	Categories	*n*	MOHi		*p*
			Mean (SD)	95% CI for mean	
Self-reported periodontitis	No	600	1.12 (0.38)	1.09–1.15	**<0.001** **
	Yes	434	1.35 (0.41)	1.31–1.39	
Sex	Female	601	1.24 (0.42)	1.21–1.27	0.066*
	Male	433	1.19 (0.39)	1.15–1.23	
Age groups	18–44	454	1.02 (0.35) ^a^	0.99–1.05	**<0.001** **
	45–64	367	1.32 (0.38) ^b^	1.28–1.36	
	65+	213	1.47 (0.36) ^c^	1.42–1.52	
Education level	Elementary	338	1.36 (0.41) ^a^	1.32–1.41	**<0.001** **
	Middle	346	1.24 (0.39) ^b^	1.20–1.28	
	Higher	350	1.06 (0.37) ^c^	1.02–1.10	
Smoking status	Never	550	1.13 (0.39) ^a^	1.10–1.16	**<0.001** **
	Former	245	1.29 (0.39) ^b^	1.24–1.34	
	Active	239	1.35 (0.42) ^b^	1.29–1.40	
Professional status	Employed	702	1.20 (0.39) ^a^	1.18–1.23	**<0.001** **
	Student	116	0.83 (0.25) ^b^	0.79–0.88	
	Retired	175	1.49 (0.34) ^c^	1.44–1.54	
	Unemployed	41	1.39 (0.36) ^c^	1.28–1.51	
Marital status	Married	466	1.30 (0.38) ^a^	1.27–1.34	**<0.001** **
	Divorced	120	1.40 (0.39) ^a,b^	1.33–1.47	
	Widowed	47	1.45 (0.40) ^b^	1.37–1.60	
	Single	401	1.04 (0.37) ^c^	1.00–1.07	

Significant *p*-values (*p* < 0.05) denoted in bold.

* t-Student's test.

** ANOVA one-way (different letters identify significant different mean MOHi values).

### 3.5 Modelling the risk profile towards a degraded oral health status based on the MOHi

The results of a multivariate logistic regression model show age, self-reported periodontitis, being female, lower education levels, and smoking were all significant predictors of degraded oral health status (MOHi ≥ 1.5) (Table 6). Active smoking and lower education emerged as the strongest risk factors, highlighting the critical role of modifiable behaviors and socioeconomic conditions in influencing oral health outcomes. The results of the reduced model for high risk of dental caries experience (DMFT ≥ 14) revealed only age, sex and smoking habits as associated risk factors (Supplementary Table S1) compared to the MOHi model.

**Table 6:** Multivariate (reduced) model (^a^) evaluating the risk towards a degraded oral health condition (MOHi >= 1.5) (*N* = 1,034).

Predictor	Categories	OR (95% CI)	*p*
Age	–	1.05 (1.04–1.06)	< 0.001
Self-reported periodontitis	No	–	–
	Yes	1.94 (1.39–2.69)	< 0.001
Sex	Male	–	–
	Female	1.80 (1.28–2.54)	< 0.001
Education level	Higher	–	–
	Middle	2.27 (1.47–3.50)	< 0.001
	Elementary	2.94 (1.91–4.54)	< 0.001
Smoking status	Never	–	–
	Former	1.62 (1.08–2.45)	0.020
	Active	3.12 (2.07–4.70)	< 0.001

a Final reduced logistic regression model obtained through a stepwise procedure; The model was statistically significant, *χ*^2^(7) = 249.978, *p* < 0.001, explained 31.9% (Nagelkerke R^2^) of the variance and correctly classified 77.9% of cases; AUC = 0.81 (95% CI: 0.78–0.84).

## 4 Discussion

This study presents the development a new combined tool aiming to classify individuals into risk categories based on a comprehensive integration of three key oral health elements: dental caries experience, OHVS, and OHRQoL. The MOHi addresses the multifaceted nature of oral health through a unified framework, and demonstrated significant correlations with age, sex, self-reported periodontitis, education level and smoking habits.

By incorporating OHV and OHRQoL into the MOHi assessment, this tool provides a more holistic understanding of oral health, capturing not only clinical disease but also its impact on patients’ lives and the values they attach to oral health. This multidimensional approach aligns with contemporary models of health that emphasize the integration of physical, psychological, and social dimensions to guide effective healthcare interventions (19, 20). Prior attempts to mix clinical indicators with patients-reported outcomes have been discussed in the past (21–23). However, these approaches lacked the OHV component, which was not introduced until its development 2021.

Since the mid-1970s, numerous instruments have been developed to investigate the impact of oral diseases on functional, social, and psychological well-being—the so-called patient-reported outcome measures (PROMs) (20, 24). Traditional oral health evaluations often focused on isolated variables, such as caries or periodontal disease, disregarding the interplay between clinical indicators and subjective experiences. Early research showed weak and inconsistent association between clinical indicators and subjective measures (24). The reduction in length of PROMs, [e.g., from OHIP-49 to OHIP-14 (5)], might facilitated stronger correlations with clinical measures (25), with relatively high burden of extensive questionnaires being a possible explanation for this (26).

Our results also highlight the potential of MOHi assessment in identifying disparities across different population groups. The inclusion of OHV and OHRQoL enables the detection of subjective vulnerabilities that might not be apparent through clinical indicators alone. For instance, individuals with low OHV scores may exhibit reduced treatment-seeking behavior despite significant clinical needs, while those with compromised OHRQoL may experience a disproportionately negative impact of oral health issues on their overall well-being. These acumens stress the potential of the MOHi assessment to inform patient-centered care strategies and targeted interventions for high-risk groups.

The MOHi assessment demonstrates considerable potential for application in both clinical practice and public health planning. In clinical settings, it may function as a screening instrument to stratify patients according to risk and prioritize interventions for those with the greatest need. For public health practitioners, the MOHi assessment could inform resource allocation and the development of tailored programs aimed at mitigating oral health disparities. Its capacity to integrate self-reported and subjective data also underscores the significance of patient engagement in oral health management, aligning with broader objectives of person-centered healthcare.

### 4.1 Strengths and limitations

While the MOHi assessment represents a promising innovation, several limitations warrant consideration. First, the self-reported components may be subject to reporting bias, potentially affecting the accuracy of risk categorization. Also, the association with self-reported periodontitis warrants due to being a modest prediction screening tool (80%) that does not replace clinical diagnosis (12). Future studies might explore whether periodontal clinical diagnosis links with MOHi remains. Second, the applicability of the MOHi assessment across diverse populations remains to be tested. Factors such as cultural differences in oral health values and access to care could influence its generalizability. Additional research is needed to refine the tool and evaluate its performance in varied contexts. Third, while the MOHi assessment provides valuable insights into risk stratification, it does not account for broader determinants of oral health, such as other oral diseases (e.g., xerostomia, temporomandibular disorder, bruxism), systemic conditions, environmental factors, and genetic predispositions. The integration of these variables into future iterations of the tool could potentially enhance its predictive capability.

## 5 Conclusion

The MOHi represents a meaningful step forward in advancing the multidimensional evaluation of oral health risk. Future research should focus on the external validation of this tool and exploring its implementation in diverse clinical and public health settings.

## Data Availability

The datasets presented in this study can be found in online repositories. The names of the repository/repositories and accession number(s) can be found below: https://doi.org/10.5281/zenodo.14794147.
