# Supplementary material for: Development and evaluation of a new multidimensional oral health indicator
Source: Front Oral Health. 2025 Sep 30;6:1634245. doi: 10.3389/froh.2025.1634245 (PMC12518264; doi:10.3389/froh.2025.1634245)
Supplement: Supplementary file 1 [file Table1.docx]

Article title:

Development and evaluation of a new multidimensional oral health indicator

# Supplementary Files

**Table S1**. Multivariate (reduced) model (*) evaluating the risk towards very high risk of dental caries experience (WHO cutoff ≥ 14) (N=1,034).

| **Predictor** | **Categories** | **OR (95% CI)** | ***p*** |
| --- | --- | --- | --- |
| Age | - | 1.08 (1.07-1.09) | < 0.001 |
| Sex | Male | - | - |
|  | Female | 1.51 (1.11-2.06) | < 0.001 |
| Smoking status | Never | - | - |
|  | Former | 1.54 (1.06-2.24) | 0.023 |
|  | Active | 2.18 (1.51-3.16) | < 0.001 |

* Final reduced logistic regression model obtained through a stepwise procedure; The model was statistically significant, χ^2^(4)=373.754, p <0.001, explained 40.7% (Nagelkerke R^2^) of the variance and correctly classified 74.8% of cases; AUC = 0.83 (95% CI: 0.80-0.85)
